# Abscisic Acid Receptors Modulate Metabolite Levels and Phenotype in *Arabidopsis* Under Normal Growing Conditions

**DOI:** 10.3390/metabo9110249

**Published:** 2019-10-24

**Authors:** Xiaoyi Li, Lintao Wu, Yao Qiu, Tao Wang, Qin Zhou, Qian Zhang, Wei Zhang, Zhibin Liu

**Affiliations:** 1 Key Laboratory of Bio-Resources and Eco-Environment of Ministry of Education, College of Life Sciences, Sichuan University, Chengdu 610065, China; 2014322040033@stu.scu.edu.cn (X.L.); QIUYAOYAO0110@163.com (Y.Q.); m15828125309@163.com (T.W.); zhouqin0116@126.com (Q.Z.); zhangchien@163.com (Q.Z.); 2 Rape Research Institute, Guizhou Academy of Agricultural Sciences, Guiyang 550008, China; wut221@126.com; 3 College of Bioengineering, Sichuan University of Science & Engineering, Zigong 643000, China; zhangwei19840117@163.com

**Keywords:** Abscisic acid (ABA), ABA receptors, ABA signaling, plant growth, metabolite profile

## Abstract

Abscisic acid (ABA) is a vital phytohormone that accumulates in response to various biotic and abiotic stresses, as well as plant growth. In *Arabidopsis thaliana*, there are 14 members of the ABA receptor family, which are key positive regulators involved in ABA signaling. Besides reduced drought stress tolerance, the quadruple and sextuple mutants (*pyr1pyl1pyl2pyl4* (1124) and *pyr1pyl1pyl2pyl4pyl5pyl8* (112458) show abnormal growth phenotypes, such as decreases in yield and height, under non-stress conditions. However, it remains unknown whether ABA receptors mediate ABA signaling to regulate plant growth and development. Here, we showed the primary metabolite profiles of 1124, 112458 and wild-type (WT) plants grown under normal conditions. The metabolic changes were significantly different between ABA receptor mutants and WT. Guanosine, for the biosynthesis of cyclic guanosine 3′,5′-monophosphate (cGMP), is an important second messenger that acts to regulate the level of ABA. In addition, other amino acids were increased in the 112458 mutant, including proline. These results, together with phenotype analysis, indicated that ABA receptors are involved in ABA signaling to modulate metabolism and plant growth under normal conditions.

## 1. Introduction

Abscisic acid (ABA) is a key hormone involved in plant growth and development, including seed dormancy and germination, root system development, leaf senescence, flowering transformation, seed and fruit ripening [1]. In addition, it also plays an important role in plant stress adaptation [1,2,3]. The core ABA signaling pathway is composed of three components. Firstly, the ABA receptors, known as the PYRABACTINRESISTANT/PYRABACTIN RESISTANT-LIKE/REGULATORY COMPONENT OF ABA RECEPTORs (PYR/PYL/RCARs, hereafter referred to as RCAR) [4,5]. Secondly, cluster A type 2C phosphatase (PP2C) family members, including ABI1, ABI2, PP2CA, HAB1, and HAB2, which are negative regulators of early ABA signaling. Finally, OST1/SnRK2.6/SnRK2E SNF1-related protein kinases 2(SnRK2s), which are important mediators. In the absence of ABA, the PP2Cs inhibit the activities of both binding and de-phosphorylating SnRK2s; when ABA is present, it binds to RCARs, which then bind to PP2Cs, thus promoting the kinase inhibition activities of SnRK2s, and simultaneously making them able to activate downstream transcription factors (ABREs) or ion channels [3,4,6].

ABA receptors constitute a 14-member family, which is found in higher plants [7,8]. The receptors are divided into three subgroups in *Arabidopsis*. Subgroup I members interact with the PP2Cs, and generate the high-affinity interaction at basal levels of ABA. All of them are able to activate ABA-responsive gene expression in protoplast transfection assays [9,10]. Whereas, most of the subgroup II members bind to PP2Cs in an ABA-independent manner [11]. Members in the subgroup III are dimeric (RCAR11, RCAR12, RCAR14), either in the presence or in the absence of ABA, showing a higher *K_d_* for ABA than monomeric ones [12]. RCAR13 is at monomer–dimer equilibrium if a monomer is present [13]. According to their different expression patterns, substantial functional differences can be expected between them. For instance, ABA specifically stabilizes PYL8 compared with other receptors and induces accumulation of PYL8 in root nuclei [14]. Given that there are 14 RCARs and 9 PP2Cs in *Arabidopsis*, functional analyses suggest that plants are able to respond to a wide range of ABA concentrations in different situations [2,4,5,15].

The *pyr1 pyl1 pyl2 pyl4* quadruple mutant (abbreviated as 1124) and the *pyr1 pyl1 pyl2 pyl4 pyl5 pyl8* sextuple mutant (112458) lost ABA receptor function. The 1124 and 112458 are insensitive to ABA, including inhibition of germination, root elongation, and impaired ABA-induced stomatal closure [4,16,17]. Until now, there has been no report on the metabolic profiles among the ABA receptors in mutant and the wild-type plants.

To further investigate if this phenotypic change was caused by disruption of ABA signaling, the two mutants and the wild-type (WT) plants were grown on agar plates with or without supplementary ABA. Metabolomics is an approach to study metabolites in seedlings for qualitative and quantitative analyses. We used UPLC-MS/MS to investigate the chemical composition of the wild-type plants, 1124 and 112458, respectively. These results might provide basic information on the ABA signaling pathway.

## 2. Results

**Figure 1:**
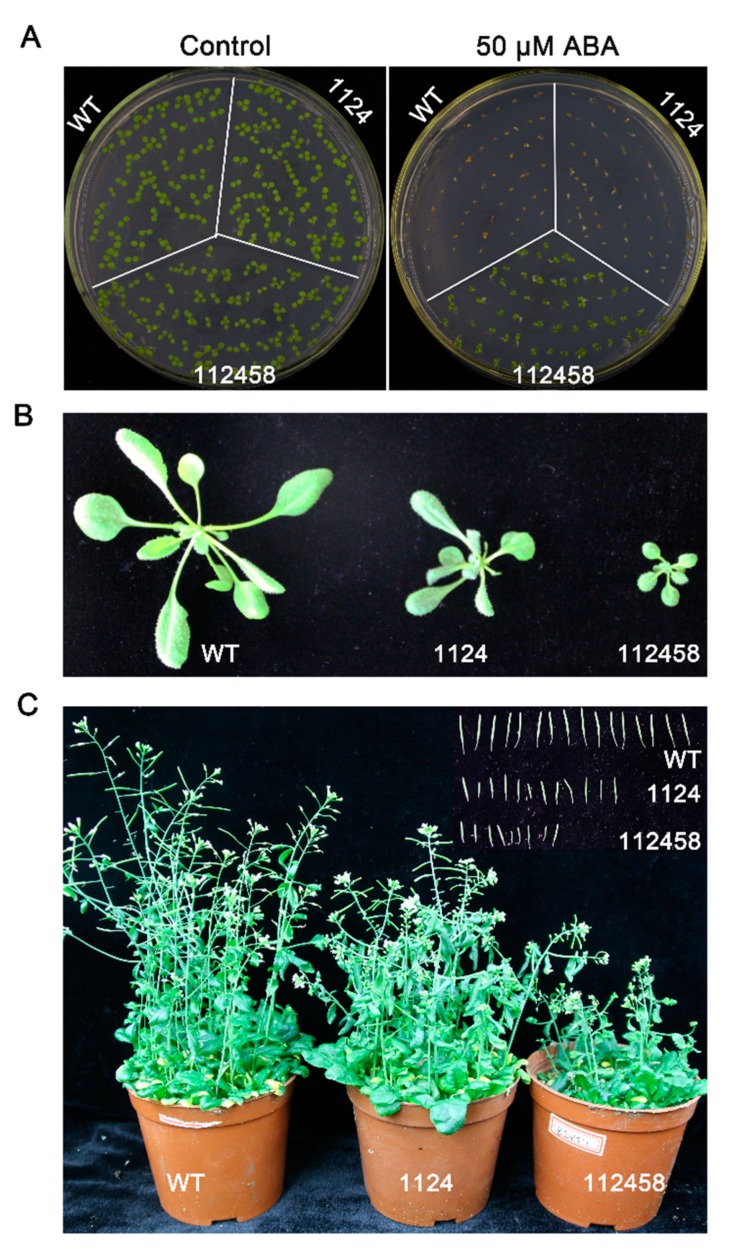
Combined loss of function of RCAR genes impairs plant growth. (**A**) Photographs of Col wild-type, 1124 and 112458 plants grown for 7 days on MS medium either lacking or supplemented with 50 μM ABA. (**B**,**C**) Photographs show the impairment of growth and reproduction in ABA receptors mutants. Photographs of 20-d-old plants (**B**), 40-d-old plants and siliques (**C**) grown under greenhouse conditions of Col wild-type, 1124 and 112458.

In order to identify the sensitivity to ABA, we detected the germination rates of the wild-type (WT), 1124 and 112458 plants, which were about 3% for WT plants, 50% for 1124 plants, and 100% for 112458 plants (Figure 1A). In addition, 112458 plants showed 100% cotyledon greening, while WT and 1124 plants did not (Figure 1A). These results are consistent with the extreme ABA-insensitive phenotype of the 112458 mutant in germination and seedling establishment assays [16]. In soil, the 1124, and particularly the 112458, mutants showed impaired growth (Figure 1B,C). Compared with the wild type, although lower growth was observed in 1124 and 112458 plants, which lost the function of binding ABA, they were able to flower and produce viable seeds (Figure 1C).

**Figure 2:**
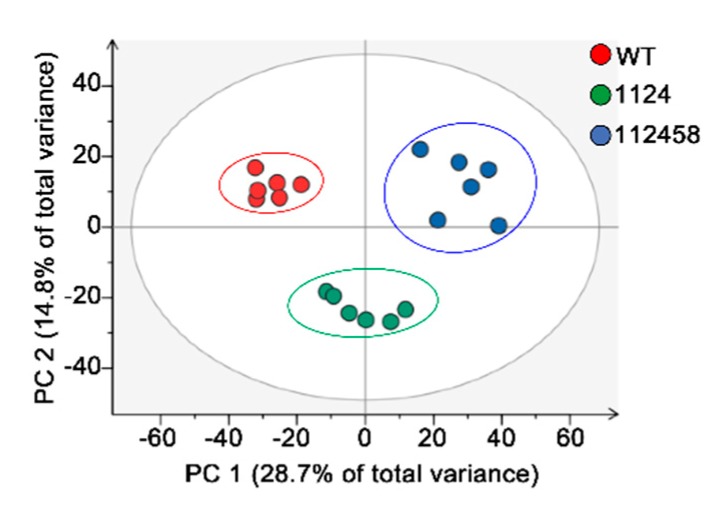
Principal component analysis of metabolite profiles of seedlings from wild-type (WT, red), 1124 (green), and 112458 (blue) plants. Each point represents an individual biological replicate. Plotting of the first and second component is shown. The circles indicate the 95% confidence regions.

**Figure 3:**
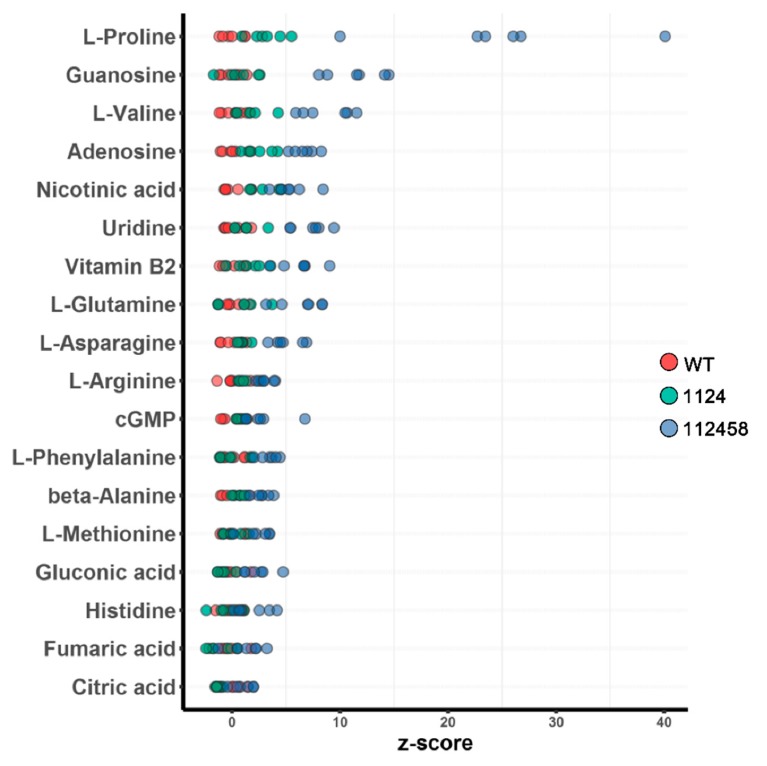
Comparison of metabolite levels in WT (red), 1124 (green), and 112458 (blue) plants.

In order to understand whether ABA signaling is a rational approach to improving crop performance under limited water supply conditions, we detected the metabolism of WT, 1124 and 112458 plants. The results showed substantial differences among them. Principal component analysis (PCA) revealed the differences in the metabolite profiles of the plants under the normal conditions (Figure 2). The metabolite levels are compared in Figure 3. The 112458 sextuple mutant had much higher levels of all 18 metabolites. A significant difference between 1124 and WT, and 112458 and WT was observed (Supplementary Table S1). In addition, the 112458 plants accumulated fumaric acid, while they did not accumulate citric acid. On the other hand, the sextuple mutant had the highest levels of amino acids including proline, guanosine, valine and adenosine. Furthermore, some metabolites remained unchanged among the WT, 1124 and 112458 plants.

**Figure 4:**
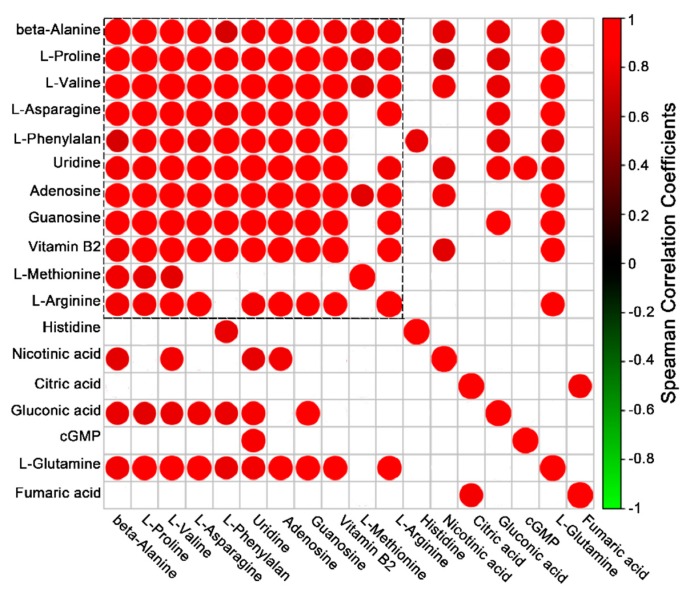
Correlation matrix and cluster analysis of the results obtained from data on 18 metabolites for WT (red), 1124 (green), and 112458 (blue) plants. Each square indicates the Pearson’s correlation coefficient for a pair of compounds, and the value for the correlation coefficient is represented by the intensity of the blue or red color as indicated on the color scale. Hierarchical clusters are represented by a cluster tree.

We performed a hierarchical cluster analysis (HCA) using Pearson’s correlation to classify the relationships between the concentrations of the 18 metabolites putatively identified from the 1124, 112458 and wild-type plants. HCA has been used as a means of assessing a relationship among quantitative samples. The HCA results showed the significantly positive correlation among the putatively identified metabolites, including beta-alanine, l-proline, l-valine, l-asparagine, uridine, adenosine, guanosine, vitamin B2, l-methionine and l-arginine (Figure 4). There was no negative correlation found among the detected metabolites. The results showed that compounds from the same class in the biological system that contains amino acids are marked by a dotted box in Figure 4.

## 3. Discussion

ABA is a vital hormone that is involved in the plant response to drought stress, playing a key role in both adapting metabolism and gene expression, at both the transcriptional and posttranscriptional levels, to help the plant cope with stress conditions [3,18]. In particular, no information is available on the metabolite shifts among the ABA receptor mutants and wild-type plants. Our main goal was to investigate the metabolic reconfiguration in the ABA signaling pathway to regulate plant growth.

ABA perception by different types of ABA receptors has been reported over the past few years [3,4,5,19,20]. In this study, we showed that impairment of ABA perception mediated by key members of the RCAR family led to a global dramatic ABA-insensitive phenotype, showing impaired growth and seed production (Figure 1), which is consistent with the assumption that basal ABA signaling is required for vegetative and reproductive growth, stomatal aperture, and transcriptional responses to the hormone [21]. *Pyl1/4/6* exhibited the best growth and improved grain productivity in rice, however, it showed earlier flowering than the wild-type plants [22]. This may be an evolutional strategy to increase productivity for survival. Another possibility is that a function of ABA receptors is to exhibit species divergence. Therefore, our results confirmed that RCAR receptors play a vital role in basal ABA signaling, which is required for plant growth even under non-stress conditions.

In order to understand whether ABA signaling is a rational approach to improving crop performance under limited water supply conditions, we analyzed the metabolism of WT, 1124 and 112458 plants under normal conditions. In addition to extreme ABA-insensitivity, the 1124 and 112458 mutants showed an abnormal phenotype under non-stress conditions (Figure 1). This observation implies that ABA signaling mediated by ABA receptors negatively regulates plant growth and the related metabolism. To investigate whether the growth phenotype of ABA receptor quadruple and sextuple mutants (1124 and 112458) is linked to changes in their metabolism, we analyzed metabolite profiles by using gas-chromatography mass-spectrometry (GC-MS). The three plants have very distant metabolic profiles, already clearly visible in the absence of stress (Figure 2, Figure 3 and Figure 4). In addition, the metabolite profile of the *nced3* (*nine-cis-epoxycarotenoid dioxygenase 3*) mutant, which is unable to synthesize ABA in response to dehydration, coupled with transcriptomic data, reveals the metabolic pathways that are regulated by ABA signaling under dehydration conditions [23]. Therefore, metabolic pathways mediate ABA signaling to regulate plant growth under stress conditions, as well as under normal conditions.

In the annotation of 18 metabolites, the relative amount of each was normalized to its level in WT plants. Subsequently, PCA revealed that the metabolite profiles of the three phenotypes were significantly different. In addition, proline, guanosine, valine and adenosine were found in high levels in the 112458 plants, whereas the amounts of other intermediates were not different compared with the wild-type plants (Figure 4 and Supplementary Table S1). These metabolites are mainly involved in arginine, proline, alanine, aspartate, glutamate, pyrimidine, pantothenate and nitrogen metabolism, as well as CoA biosynthesis, and the TCA cycle. Arginine, a conditionally essential amino acid, is synthesized from citrulline in arginine and proline metabolism in the cytosol [24]. The previous study showed that nitric oxide (NO) may inhibit ABA signaling through inactivation of ABA receptors by tyrosine nitration [25], which is consistent with detected metabolites involving in the nitrogen metabolism. These results are consistent with previous reports that the TCA cycle can operate in non-cyclic flux modes in plants [26,27]. The citrate levels did not change among the 1124, 112458 and wild-type plants, which is opposite to the trend in the *srk2d srk2e srk2i* mutant and WT plants during seedling growth [28]. On the other hand, SnRK2s are responsible for maintaining normal metabolism and leaf growth under non-stress conditions, which is consistent with the fact that the receptors are required for maintaining plant growth (Figure 1). These may be due to other molecules that contribute to the different metabolite profiles among the 1124, 112458 and *srk2d srk2e srk2i* mutants, such as reactive oxygen species (ROS), which are short-lived molecules in response to abiotic stimulus [29]. The *Arabidopsis* proline level is involved in development and abiotic stress tolerance [28,30]. The 112458 mutant showed significantly higher levels of proline than the WT plants, which is consistent with the assumption that loss of ABA receptors would cause severe stress tolerance. Furthermore, the ABA signaling pathway also overlaps with Pro biosynthesis and signaling, contributing to the redox balance of the cell under normal and stress conditions [31].

In conclusion, the plants respond to stress conditions through the ABA signaling pathway, which also mediates amino acid biosynthesis under normal conditions.

## 4. Materials and Methods

### 4.1. Plant Materials and Growth Conditions

*Arabidopsis thaliana* plants were grown under greenhouse conditions (40–50% relative humidity) in pots containing a 1:3 vermiculite: soil mixture. For plants grown under growth chamber conditions, seeds were surface sterilized with 1% commercial bleach (NaOCl) (*v*/*v*) for 10 mins and grown on plates with Murashige and Skoog medium plus 2% sucrose and 0.8% agar at 22 °C under a long-day (16-h light/8-h dark) photoperiod at 80 to 100 μEm^−2^s^−1^. The 1124 and 112458 mutants have been described previously [16].

### 4.2. Seedling Establishment Assay

After surface sterilization of the seeds, stratification was conducted in the dark at 4 °C for 3 d. Next, about 50 seeds of each genotype were sown on MS plates supplemented with 50 μM ABA. Seedling establishment was photographed at 7 d after sowing.

### 4.3. Metabolite Extraction and Sample Preparation for Metabolomics

Each independent preparation was regarded as a replicate. A total of six replicates, each of 100 mg lyophilized sample were sent to Smart Nuclide (Suzhou, China) for UPLC (Water, MilfordMA, USA, ACQUITY UPLC) and MS (Thermo, LTQ Oritrap XL)-based metabolomics analyses. The samples were extracted with 250 μL methanol aqueous solution (1:1, 4 °C), and filtered using 0.22 μm membrane filters before LC-MS detection. Chromatographic separation was accomplished in an Acquity UPLC system equipped with an ACQUITY UPLC^®^ HSS T3 (150 × 2.1 mm, 1.8 μm, Waters, MilfordMA, USA) column maintained at 40 °C. The temperature of the autosampler was 4 °C. Gradient elution of analytes was carried out with 0.1% formic acid in water (A) and 0.1% formic acid in acetonitrile (B) at a flow rate of 0.25 mL/min. Injection of 3 μL of each sample was done after equilibration. An increasing linear gradient of solvent B (*v*/*v*) was used as follows: 0–1 min, 2% B; 1–9.5 min, 2–50% B; 9.5–14 min, 50–98% B; 14–15 min, 98% B; 15–15.5 min, 98–2% B; 15.5–17 min, 2%. The ESI-MS^n^ experiments were executed on the Thermo LTQOrbitrap XL mass spectrometer with the spray voltage of 4.8 kV and −4.5 kV in positive and negative modes, respectively. Sheath gas and auxiliary gas were set at 40 and 15 arbitrary units, respectively. The capillary temperature was 325 °C. The voltages of capillary and tube were 35 V and 50 V, and −15 V and −50 V in positive and negative modes, respectively. The Orbitrap analyzer scanned over a mass range of *m*/*z* 50–1000 for full scan at a mass resolution of 60,000. Data dependent acquisition (DDA) MS/MS experiments were performed with CID scan. The normalized collision energy was 30 eV. Dynamic exclusion was implemented with a repeat count of 2, and exclusion duration of 15 s.

The National Institute of Standards and Technology were used to identify the metabolite. The results were filtered with retention time, quality control, and mass spectral matching by Proteowizard (v3.0) and the program XCMS of R (v3.1.3). As a result, a total of 18 metabolites were putatively identified. Concentration calculations of all metabolites were based on the ratios determined from the peak area of each metabolite/the peak area of the IS.

### 4.4. Metabolomics Data Processing and Statistics

The data processing method was described in detail in a previous publication [32,33]. Quantification data obtained from UPLC-MS were performed to PCA and PLS-DA (partial least squares-discriminant analysis, SIMCA-P (v13.0); Umetrics, Umea, Sweden) to assess the similarity between groups of data. Differentially changed metabolites were analyzed using R-based empirical analysis. Raw data were uploaded to the MetaboAnalyst 3.0 webserver and only metabolites mapped to the KEGG pathway database were used. A log_2_ transformation was used to normalize and scale the data before further analysis.

## Supplementary Materials

The following are available online at https://www.mdpi.com/2218-1989/9/11/249/s1. Table S1: A significant difference between 1124 and WT, and 112458 and WT was observed.

## Abbreviations

ABA	abscisic acid
WT	wild-type *Arabidopsis thaliana*
1124	*pyr1pyl1pyl2pyl4*
112458	*pyr1pyl1pyl2pyl4pyl5pyl8*
PYR/PYL/RCARs	PYRABACTINRESISTANT/PYRABACTIN RESISTANT-LIKE/REGULATORY COMPONENT OF ABA RECEPTORs
GC-MS	Gas chromatography-mass spectrometry
